# Temporal Regulation of the *Bacillus subtilis* Acetylome and Evidence for a Role of MreB Acetylation in Cell Wall Growth

**DOI:** 10.1128/mSystems.00005-16

**Published:** 2016-05-31

**Authors:** Valerie J. Carabetta, Todd M. Greco, Andrew W. Tanner, Ileana M. Cristea, David Dubnau

**Affiliations:** a Public Health Research Center at New Jersey Medical School, Rutgers University, Newark, New Jersey, USA; b Department of Molecular Biology, Princeton University, Princeton, New Jersey, USA; University of British Columbia

**Keywords:** lysine acetylation, MreB, acetylome, cell wall synthesis, label-free quantification, mass spectrometry, peptidoglycan, posttranslational modification, proteomics

## Abstract

*N*^ε^-Lysine acetylation has been recognized as a ubiquitous regulatory posttranslational modification that influences a variety of important biological processes in eukaryotic cells. Recently, it has been realized that acetylation is also prevalent in bacteria. Bacteria contain hundreds of acetylated proteins, with functions affecting diverse cellular pathways. Still, little is known about the regulation or biological relevance of nearly all of these modifications. Here we characterize the cellular growth-associated regulation of the *Bacillus subtilis* acetylome. Using acetylation enrichment and quantitative mass spectrometry, we investigate the logarithmic and stationary growth phases, identifying over 2,300 unique acetylation sites on proteins that function in essential cellular pathways. We determine an acetylation motif, EK(ac)(D/Y/E), which resembles the eukaryotic mitochondrial acetylation signature, and a distinct stationary-phase-enriched motif. By comparing the changes in acetylation with protein abundances, we discover a subset of critical acetylation events that are temporally regulated during cell growth. We functionally characterize the stationary-phase-enriched acetylation on the essential shape-determining protein MreB. Using bioinformatics, mutational analysis, and fluorescence microscopy, we define a potential role for the temporal acetylation of MreB in restricting cell wall growth and cell diameter.

**IMPORTANCE** The past decade highlighted *N*^ε^-lysine acetylation as a prevalent posttranslational modification in bacteria. However, knowledge regarding the physiological importance and temporal regulation of acetylation has remained limited. To uncover potential regulatory roles for acetylation, we analyzed how acetylation patterns and abundances change between growth phases in *B. subtilis*. To demonstrate that the identification of cell growth-dependent modifications can point to critical regulatory acetylation events, we further characterized MreB, the cell shape-determining protein. Our findings led us to propose a role for MreB acetylation in controlling cell width by restricting cell wall growth.

## Abstract (precis)

The past decade highlighted *N*^ε^-lysine acetylation as a prevalent posttranslational modification in bacteria. However, knowledge regarding the physiological importance and temporal regulation of acetylation has remained limited. To uncover potential regulatory roles for acetylation, we analyzed how acetylation patterns and abundances change between growth phases in *B. subtilis*. To demonstrate that the identification of cell growth-dependent modifications can point to critical regulatory acetylation events, we further characterized MreB, the cell shape-determining protein. Our findings led us to propose a role for MreB acetylation in controlling cell width by restricting cell wall growth.

## INTRODUCTION

The physiological importance of *N*^ε^-lysine acetylation as a regulatory posttranslational modification (PTM) is emphasized by the association of its dysregulation with heart disease and aging (1), obesity and diabetes (2), Alzheimer’s disease (3), and certain cancers (4). The level of protein acetylation is regulated by the opposing actions of lysine acetyltransferases (KATs) and deacetylases (KDACs). The best understood, evolutionarily conserved family of KATs contains the GCN5-like acetyltransferases (GNATs), which catalyze the transfer of an acetyl group from acetyl coenzyme A (acetyl-CoA) to a lysine primary amine (5, 6). The deacetylation reaction may be carried out by Zn^+^-dependent lysine deacetylases (KDACs) or by NAD^+^-dependent sirtuins (7). Acetylation has been shown to affect protein activity (8), protein-protein or protein-DNA interactions (9, 10), local protein conformation (11), and subcellular localization (12, 13).

The advancement of highly accurate and higher-resolution mass spectrometry (MS) techniques combined with the improved specificity of anti-acetyl lysine antibodies has allowed for in-depth, global characterization of cellular acetylomes. Global eukaryotic acetylome studies (14–21) have found that the mitochondrial proteome is highly acetylated, representing nearly 20% of its predicted proteome (21–23). The evolutionary relationship between mitochondria and bacteria has prompted large-scale investigations of acetylation in both Gram-negative (24–35) and Gram-positive (36–43) bacteria. Recently, it has been discovered that acetylation in bacteria is ubiquitous, with hundreds of acetylated proteins potentially affecting many diverse cellular processes.

Despite this growing number of known acetylated proteins in bacteria, the understanding of their function or regulation remains limited. The best-characterized regulatory acetylation in bacteria controls the activity of acetyl-CoA synthetase (AcsA) (31, 44–50). In *Bacillus subtilis*, AcsA is acetylated at a conserved lysine residue within its active site by the KAT enzyme AcuA (51). When increased acetyl-CoA production is required, AcsA is deacetylated by the KDAC homolog AcuC (45) and the sirtuin SrtN (52) and becomes activated. Thus, the activity of AcsA is finely tuned to the levels of acetyl-CoA and NAD^+^. Interestingly, this mechanism is evolutionarily conserved, even in human mitochondria (31, 44–50).

Functional roles of other specific acetylation events have not been characterized in *B. subtilis*, which is the best-studied Gram-positive bacterium. Moreover, global analysis of the *B. subtilis* acetylome has thus far been performed at a single time point during stationary-phase growth in rich medium (38) or in media with alternate carbon sources (43). Here, we have characterized the lysine acetylome during both the logarithmic and stationary phases. A quantitative mass spectrometry-based proteomics approach was used to measure temporal changes in protein abundance and acetylation at specific lysine residues. Qualitatively, we have identified acetylation on proteins that cover ~20% of the *B. subtilis* proteome. The identified acetylation sites point to a motif with the core sequence EK(ac)(D/Y/E), in agreement with other bacterial species (24, 27–29, 32, 35, 36, 38, 40, 41, 43) and human mitochondria (14), suggesting conserved regulatory mechanisms. Bioinformatic analysis supports the potential role of acetylation in growth stage-specific regulation of protein function. Based on our differential acetylome analysis, we conducted a functional analysis of the essential cell shape-determining protein MreB, which exhibited a stationary-phase-specific increase in acetylation at a single lysine residue. This characterization suggested a contribution of MreB acetylation in regulating cell wall growth.

## RESULTS

### Lysine acetylation is prevalent in *B. subtilis* and temporally regulated throughout growth.

**FIG 1:**
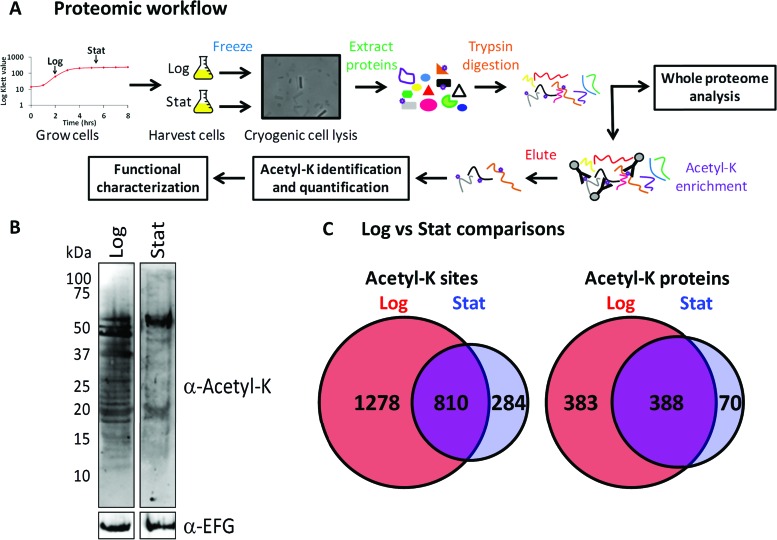
Acetylation is a dynamic modification in *B. subtilis*. (A) Wild-type cells (BD630) were grown to the log or stat phase in minimal glucose medium. Arrows on the growth curve indicate the mid-log-phase and early-stat-phase time points used for all studies. Cells were harvested and rapidly frozen for cryogenic cell lysis. This technique was used to obtain a more consistent disruption of the cells, which leads to improved efficiency of protein extraction and reproducibility of acetyl-peptide capture. The image depicts a sample after grinding showing nearly complete cell lysis (>85%). Lysed cell material was resuspended in a heated SDS buffer, and proteins were precipitated and subjected to digestion with trypsin. An aliquot of peptides was saved for whole-cell protein analysis, while the remaining peptides were incubated with agarose beads coated with a mixture of commercially available anti-acetyllysine antibodies. Acetyllysine peptide elutions and peptides from the whole-cell digests were analyzed by label-free quantitative mass spectrometry using nanoscale liquid chromatography (nLC) coupled directly to an LTQ Orbitrap Velos ETD mass spectrometer. Peptide MS/MS sequencing was performed for both sample sets. (B) Wild-type cells were grown to the log and stat phases and lysates prepared as described in Materials and Methods. Equal amounts of protein were loaded in duplicate, with one set probed with a mixture of anti-acetyllysine (acetyl-K) antibodies and the other probed with anti-elongation factor G (anti-EFG) as a loading control. Log- and stat-phase samples were analyzed on the same membrane. (C) Venn diagrams comparing the number of identified acetylated sites (left) and proteins (right) in the log (red) and stat (blue) phases.

To characterize the *B. subtilis* acetylome and gain insight into the potential significance of acetylation events, we monitored changes in protein acetylation patterns and abundance. We chose to characterize the dynamic changes occurring during logarithmic (log)- and stationary (stat)-phase growth, because differential acetylation of lysine residues might occur during rapid growth and be of particular relevance for cells progressing from the log into the stat phase. Wild-type cells were grown in minimal glucose medium, and samples were taken for analysis by immunoblotting with antiacetyllysine antibodies (Fig. 1A, growth curve, indicated by arrows). A striking difference was observed, with prevalent global acetylation during the log phase and a dramatic decrease by the early stat phase (Fig. 1B). To measure changes in lysine acetylation at the level of specific proteins and lysine residues, we designed a mass spectrometry (MS)-based proteomic work flow (Fig. 1A). Isolated acetylated peptides were analyzed by mass spectrometry in three independent biological replicates and two technical replicates. Global proteome changes were also monitored by mass spectrometry at each growth phase to determine whether changes in acetylation corresponded to changes in PTM stoichiometry or overall protein abundance.

We identified 2,372 unique sites on 841 proteins, accounting for ~20% of the predicted proteome (see Table S1 and Fig. S1 in the supplemental material). Of these acetylation sites, 2,088 were detected in the log phase and 1,094 in the stat phase (Fig. 1C). Therefore, mass spectrometry analysis confirmed the global reduction in stat-phase acetylation observed by Western blot analysis of whole-cell lysates (Fig. 1B). As expected, this reduction in the number of sites was accompanied by a reduction in the overall number of acetylated proteins (Fig. 1C; 771 log- and 458 stat-phase proteins). These data demonstrate extensive protein acetylation that is temporally changed depending on growth state. Gene Ontology (GO) analysis (53) of the acetylated proteins showed a significant enrichment of proteins involved in translation, including ribosomal proteins and elongation factors, and enzymes involved in intermediary metabolism (see Fig. S2 in the supplemental material).

10.1128/mSystems.00005-16.8 Table S1  List of all identified acetylated peptides and label-free quantification. Download Table S1, XLSX file, 0.6 MB. Copyright © 2016 Carabetta et al. 2016 Carabetta et al. This content is distributed under the terms of the Creative Commons Attribution 4.0 International license.

10.1128/mSystems.00005-16.1 Figure S1  Identification and matching of acetyl-K peptides across biological triplicates. The number of unique acetyl-K sites is shown for each of the three biological replicates and the corresponding average ± standard deviation. Unique acetyl-K peptides are reported for sequences that were identified by MS/MS only (left) or identified and matched between runs (right). Matching between runs was performed by MaxQuant to decrease missing values resulting from the random sampling effects of data-dependent acquisition. Download Figure S1, EPS file, 1.4 MB. Copyright © 2016 Carabetta et al. 2016 Carabetta et al. This content is distributed under the terms of the Creative Commons Attribution 4.0 International license.

10.1128/mSystems.00005-16.2 Figure S2  Significantly enriched GO biological processes and comparative analyses of acetylated proteins. (A) Acetylated proteins were analyzed using PANTHER GO analysis. Functional annotations are statistically overrepresented versus the entire *Bacillus subtilis* genome (corrected *P* values). Fractions represent acetylated proteins with annotation/total number of proteins in that annotation. Displayed are the top 3 categories for protein class, pathways, and biological processes. The enrichment of these particular functional classes is largely consistent with the other bacterial acetylome studies (24–30, 32, 33, 36–41, 110) and with acetylome studies of protozoa, yeast, flies, plants, rodents, and human mitochondria (17–19, 22, 23, 67, 68, 70, 111, 112). These observations suggest that lysine acetylation may be an important evolutionarily conserved regulatory mechanism for these particular classes of proteins. (B) Frequency distribution of acetylated proteins based on the number of acetylated lysine sites per protein. (C) Scatter plot showing the distribution of the number of acetylated lysines versus the total number of lysine residues per protein in the stat phase. (D) Comparison of acetyllysine sites per protein versus protein copy number estimates for stat-phase growth. Protein abundances were expressed as average ± standard deviation (top) or individual (bottom) values, which were derived from label-free MS analysis. The observed positive association between protein abundance and likelihood of acetylation is illustrated by examination of the data. For example, ribosome recycling factor (Frr) is present at about 10,000 copies per cell and contains only 21 lysine residues, but 62% of them (13 sites) were found to be acetylated. In contrast, polyketide synthase (PksN), present at <50 copies per cell, contains 353 lysines, but only one acetylation was detected. We also observed differences in the number of acetylated sites for proteins with similar abundances. The phosphoribosylformylglycinamidine synthase subunit (PurL) and glutamate synthase [NADPH] (GltB), which are each present at about 6,600 copies per cell, have one and nine identified acetylation sites, respectively (see Table S1 and S2 in the supplemental material). These observed differences in protein abundance suggest that there are specificity determinants for protein acetylation and that additional regulatory mechanisms exist beyond protein abundance. Download Figure S2, EPS file, 2.6 MB. Copyright © 2016 Carabetta et al. 2016 Carabetta et al. This content is distributed under the terms of the Creative Commons Attribution 4.0 International license.

### Protein properties that influence the presence of acetylation in *B. subtilis*.

**FIG 2:**
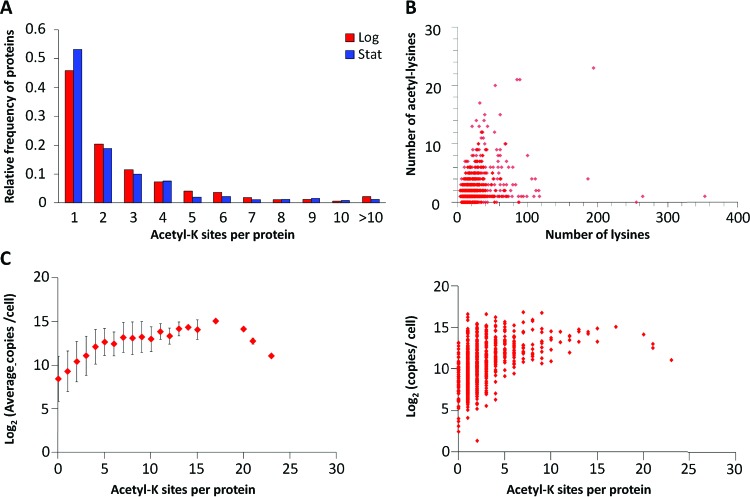
Comparison of the lysine acetylome in the log versus stationary phase. (A) Relative frequency distribution of acetylated proteins normalized to the total number of proteins in each growth phase compared to the number of acetylation sites per protein. (B) Scatter plot showing the distribution of the number of acetylated lysines versus the total number of lysine residues per protein in the log phase. (C) Comparison of acetyllysine sites per protein versus protein copy number estimates for log-phase growth. Protein abundances were expressed as average ± standard deviation (left) or individual (right) values, which were derived from label-free MS analysis.

To further examine the regulation of these acetylation events, we investigated the protein properties that may influence the presence of acetylation. We first assessed the distribution of the number of acetylation sites per protein. Similar patterns were observed for both growth phases (χ^2^ test for independence, *P* = 0.2369), with roughly half of the total proteins identified in each phase containing a single acetyllysine modification (Fig. 2A; see Fig. S2B in the supplemental material). The overall number of lysine residues per protein does not appear to influence the distribution of acetylation events for either log- or stat-phase cells, as only a weak correlation was observed between the number of acetylated sites and the total number of lysine residues in each protein (Spearman correlation coefficient [*r_s_*] = 0.1501 and 0.2134, respectively) (Fig. 2B; see Fig. S2C). Thus, the likelihood of identifying an acetylation event is not strictly a function of the number of lysine residues in a given protein, suggesting that there are specific acetylation determinants.

To systematically examine the relationship between protein abundance and the number of identified acetyllysine sites, we used MS1-based quantification of total protein abundance (see Table S2 in the supplemental material) to calculate protein copy numbers (protein molecules per cell). Using this approach, the number of identified acetylation modifications as a function of the average protein abundance clearly showed a positive association for the log (*r_s_* = 0.5443) and stat (*r_s_* = 0.5950) phases (Fig. 2C, left; see Fig. S2D, top, in the supplemental material). Indeed, we observed that many of the proteins identified with multiple acetylation sites were highly abundant proteins (54). However, the range of protein abundances for defined numbers of acetylation sites was large, particularly for those with a lower number of sites (Fig. 2C, right; see Fig. S2D, bottom). For example, proteins that contained zero or one acetylated lysine spanned the widest abundance range, from <50 copies/cell to >60,000 copies/cell. Conversely, no low-abundance proteins were identified with a large number of acetylated sites (>5 sites) (Fig. 2C, right; see Fig. S2D, bottom). Overall, from these comparisons, there is clearly a protein abundance-dependent component to the identification of the number of acetylated sites, while the number of lysine residues in a protein was less important.

10.1128/mSystems.00005-16.9 Table S2  List of all proteins detected during log- and stat-phase growth and label-free quantification. Download Table S2, XLSX file, 1 MB. Copyright © 2016 Carabetta et al. 2016 Carabetta et al. This content is distributed under the terms of the Creative Commons Attribution 4.0 International license.

### Distinct signature acetylation motifs are present during the log and stat growth phases, pointing to temporal regulation.

**FIG 3:**
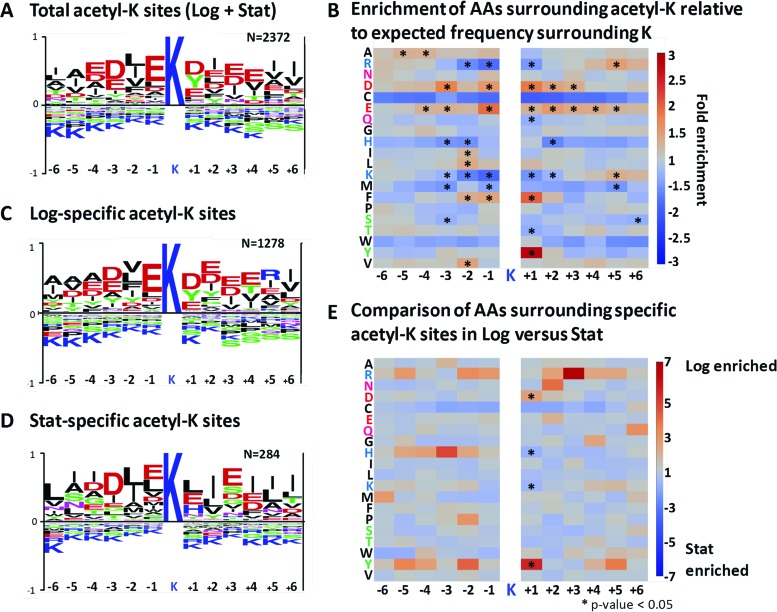
Primary sequence analysis of acetylated protein motifs reveals growth-phase-dependent modification motifs. Sequence windows (±6 residues) surrounding the identified acetylated lysine were analyzed to generate a sequence logo for (A) the entire data set (*n* = 2,372), (C) the log-specific acetylation sites (*n* = 1,278), and (D) the stat-specific (*n* = 284) acetylation sites. (B) Heat map illustrating the fold enrichment of amino acid occurrence for the acetyllysine sequences in panel A relative to the expected amino acid occurrence surrounding all lysine residues in the *B. subtilis* proteome. Statistically significant (Bonferroni corrected *P* values of <0.05) fold enrichment values are indicated by asterisks. (E) Same as panel B, except fold change in amino acid occurrence was compared between log-specific (C) and stat-specific sites (D). The uniform underrepresentation of cysteine did not reach significance because this residue is present at such a low frequency within the *Bacillus* proteome that the differences did not reach significance.

To investigate the propensity of lysines to undergo acetylation, we examined whether a pattern could be deduced from the primary sequence surrounding the acetylation site. The most prominent sequence motif displayed a preference for acidic residues, accompanied by the underrepresentation of basic residues and the frequent presence of a tyrosine residue at the +1 position (Fig. 3A). The enrichment of acidic residues was significantly overrepresented compared to the overall frequency distribution (Bonferroni-corrected *P* value of <0.05) (Fig. 3B). In addition to acidic residues, tyrosine and phenylalanine were significantly enriched at the +1 position, with tyrosine showing a markedly increased frequency of approximately 3-fold over expectation. Additionally, basic residues (histidine, lysine, and arginine) were significantly underrepresented. These features may represent a recognition sequence for regulatory enzymes, or the local environment may make the target lysine residue a preferred substrate for a nonenzymatic acetylation mechanism (see Discussion).

To determine if acetylation selectivities were different between the log and stat phases, we next analyzed separately the 1,278 and 284 acetylation sites uniquely identified in the log and stat phases, respectively. While the stat-phase-specific set of acetyllysine sites represents a small subpopulation, previous studies have used similar or smaller numbers to identify possible motifs (24, 27–29, 38–41). Although the log-phase-specific motif closely matched the overall acetylation motif (Fig. 3C versus A), the stat-phase-specific motif slightly diverged from the overall motif (Fig. 3D). Significant differences were found at the +1 position (Fig. 3E): tyrosine and aspartate were strongly favored in the log phase, while basic amino acids, histidine and lysine, were observed in the stat phase (Fig. 3E). In contrast, when the shared log- and stat-phase sites were included, motif analysis yielded results similar to the overall sequence motif (see Fig. S3 in the supplemental material). The identification of a potential acetylation signature for the stat phase points to a possible mechanism for the growth stage regulation of this posttranslational modification.

10.1128/mSystems.00005-16.3 Figure S3  Analysis of log- and stat-phase acetyl-K sequences shows no significant differences in acetylation motifs. The sequence window (±6 residues) surrounding the acetylated lysine was analyzed in order to generate sequence logos for (A) total log-phase (*n* = 2,088) and (B) total stat-phase (*n* = 1,094) sites (including sequences identified in common between conditions), using the PhosphoSite motif and logo analysis tool. (C) Heat map comparing the fold changes in amino acid (AA) occurrence between total log-phase (A) and total stat-phase (B) acetylated sites. No statistically significant changes were observed (*P* > 0.05). Download Figure S3, PDF file, 2.5 MB. Copyright © 2016 Carabetta et al. 2016 Carabetta et al. This content is distributed under the terms of the Creative Commons Attribution 4.0 International license.

### Signature changes in acetylation during the log- to stat-phase transition include proteins modulating cell structure and morphogenesis.

As changes in total protein abundances are primary determinants of cellular function, we next determined the subset of proteins with altered abundances in the stat versus log phase. We identified only 226 proteins that were significantly altered in abundance as cells transitioned from the log to stat phase. DNA-mediated transformation proteins were upregulated, as expected (55), and proteins involved in translation were downregulated (see Fig. S4 and S5 in the supplemental material). Overall, these relatively limited alterations in protein abundance suggest that the identified changes in acetylation are not due to large-scale shifts in proteome composition but rather to the modification stoichiometry.

10.1128/mSystems.00005-16.4 Figure S4  Log- to stationary-phase growth transition induces selective proteome abundance changes. (A) Volcano plot depicting the average log_2_ fold change in total protein expression (*n* = 1,947 proteins) versus the corrected *P* value for stat- versus log-phase growth. Relative abundances were measured in biological triplicates by MS-based label-free quantification. Differentially expressed proteins (Benjamini-Hochberg false discovery rate [FDR], <0.05) are represented by orange crosses. We identified 226 out of the total of 1,947 proteins that were quantified to be significantly altered in abundance (FDR, <0.05). (B and C) STRING interaction networks representing proteins assigned to biological process clusters from the Gene Ontology (GO) analysis platform ClueGO (102) (see Fig. S5 in the supplemental material) that were most significantly increased (B [DNA-mediated transformation]) or decreased (C [translation]). MS-based quantitative measurements were overlaid on the networks to reflect relative (node color) and absolute (bar charts) abundance between stat- and log-phase growth. Relative quantification was expressed as log_2_ fold change (FC [stat/log]). Absolute quantitation was expressed as estimated log_2_ copy numbers per cell in the log (blue) or stat (yellow) phase. Edge thickness indicates the interaction confidence score (0.4 to 1.0). Given that *Bacillus* cells become competent for transformation in minimal medium supplemented with glucose (55), this induction of proteins required for transformation served as a positive control. In fact, the majority of the proteins within this DNA transformation cluster, including the master regulator of competence development ComK (113), were increased several orders of magnitude. In contrast, the translation and metabolic clusters were underrepresented in the stat phase, corresponding with downregulation of ribosomal and general stress response proteins. Even though there are different metabolic and biosynthetic demands between rapid (log-phase) growth and stat-phase growth, almost 90% of the total proteins quantified had nonsignificant changes in abundance. Download Figure S4, PDF file, 2.6 MB. Copyright © 2016 Carabetta et al. 2016 Carabetta et al. This content is distributed under the terms of the Creative Commons Attribution 4.0 International license.

**FIG 4:**
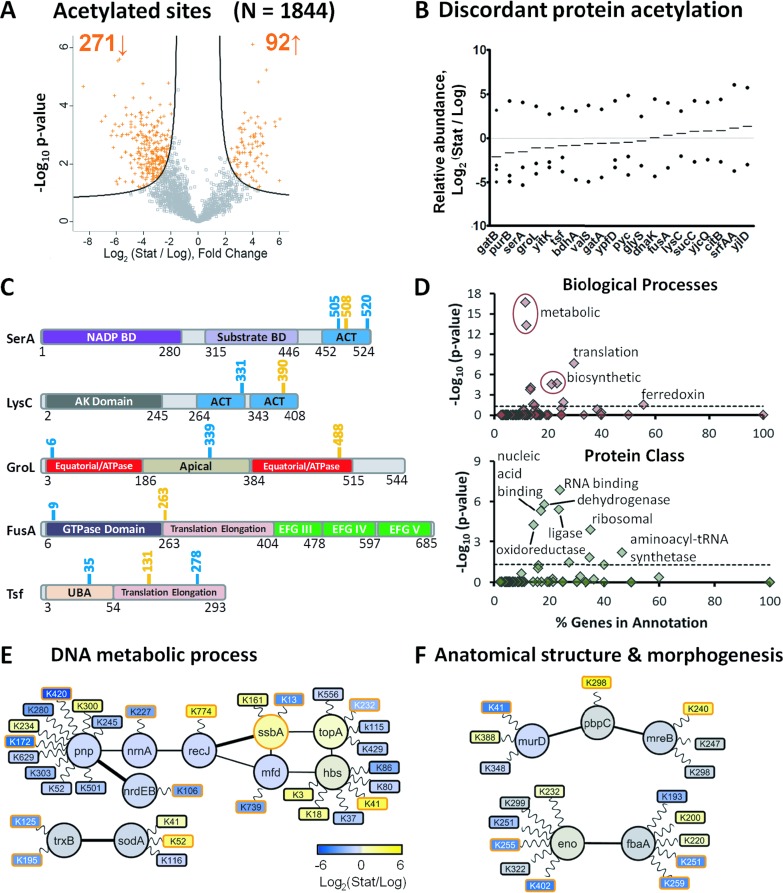
Log- to stationary-phase growth results in differential acetyl abundance on specific proteins. (A) Volcano plot depicting the average site-specific log_2_ fold change in levels of acetyllysine (*n* = 1,844 sites). Relative abundances were measured in biological triplicates by MS-based label-free quantification. Acetylation sites with statistically significant differences in abundance (Benjamini-Hochberg FDR, <0.05) are represented by orange crosses. (B) The relative change in acetylation levels of specific acetyllysine sites (filled circles) is shown for the 20 proteins that had at least one discordant site of acetylation. Proteins are sorted by increasing change in the average protein acetylation (horizontal bars). (C) Domain schematics of selected proteins with discordantly regulated acetylation sites. Acetylated residues are denoted by amino acid positions and color coded for downregulated (blue) or upregulated (yellow) relative change in the stat versus log phase. Protein domains were labeled with InterPro terms obtained from UniProtKB. Abbreviations: BD, binding domain; ACT, aspartate kinase, chorismate mutase, and TyrA; AK, aspartate kinase; EFG, elongation factor G; UBA, ubiquitin-associated domain. (D) Gene Ontology annotation and overrepresentation analysis of proteins with differential acetylation. UniProt accession numbers were annotated to PANTHER GO Slim biological processes and protein classes. For each GO term, the percentage of the total annotated genes/proteins that were found differentially acetylated is indicated on the *x* axis. This was compared on the *y* axis to the corresponding statistical significance of overrepresented acetyl proteome gene functions versus the entire genome. The dashed line reflects a Bonferroni-corrected *P* value (*P* < 0.05). Selected GO terms with the greatest statistical overrepresentation are labeled. (E and F) STRING functional relationships are visualized (solid edges) among the differentially acetylated proteins (circle nodes) in the DNA metabolic process (E) and anatomical structure morphogenesis (F) ClueGO clusters (see Fig. S7 in the supplemental material), along with each protein’s corresponding acetylated lysine residue(s) (rectangle nodes). Relative total protein and site-specific acetylation abundance changes are indicated by color scale. Statistically significant abundance changes (see Table S1 in the supplemental material) are indicated by orange node edges.

10.1128/mSystems.00005-16.5 Figure S5  GO biological process (BP) cluster comparison of differentially expressed proteins between the stat and log phases. Comparative GO analysis was performed using the ClueGO Cytoscape plugin (version 2.1.7) to visualize the biological processes (GO BP 03-10-2015) that were more representative by upregulated (red nodes) or downregulated (green) proteins in the stat phase (*n* = 226 [Fig. 4A]). Greater representation of a particular GO BP term was defined as at least 60% of the proteins assigned to that term being either up- or downregulated. Gray nodes reflect GO biological processes that were equally represented by up- and downregulated proteins. Functional clusters of biological processes are labeled with the biological process term with the smallest term *P* value. Download Figure S5, PDF file, 2.8 MB. Copyright © 2016 Carabetta et al. 2016 Carabetta et al. This content is distributed under the terms of the Creative Commons Attribution 4.0 International license.

After establishing the growth phase-dependent changes in total protein, we next evaluated site-specific changes in acetyllysine levels. We found that 271 acetylation sites were significantly downregulated in the stat phase and 92 were upregulated (false discovery rate [FDR], <0.05) (Fig. 4A). The majority of these proteins (*n* = 210) contained a single acetylation site. Interestingly, most of the proteins that contained multiple acetylations (*n* = 63) had sites that were altered in the same direction, meaning that the individual sites were either all up- or downregulated. Yet, one-third of these proteins (*n* = 20) had at least one site that was quantitatively discordant (Fig. 4B). From this subset, the total abundance of all proteins (except SrfAA) remained relatively unchanged from the log to stat phase (see Table S2 in the supplemental material). This implies that the changing acetylation patterns on these particular proteins were not due to increased protein abundance but rather were subject to other regulatory mechanisms. To investigate if the quantitative discordance could be linked to the regulation of protein function; we mapped the differential acetyl sites to known structural features. Indeed, many of these acetylation sites were contained within known functional domains (Fig. 4C; see Fig. S6 in the supplemental material), including nucleotide or substrate binding sites and regulatory domains. While this analysis did not reveal the reason for discordant regulation, the identification of differential acetylation sites within functional domains suggests the possibility that their regulation may be finely tuned by acetyllysine stoichiometry.

**FIG 5:**
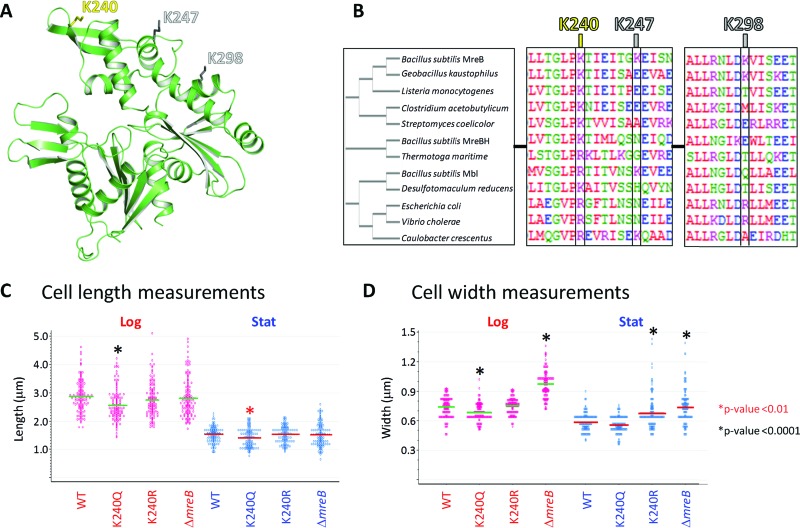
MreB lysine 240 is surface exposed, conserved, and critical for regulating cell size. (A) A ribbon X-ray crystallography model of the *B. subtilis* MreB was generated using the *C. crescentus* structure (PDBe accession no. 4cze; 59% identity) as a template in SWISS-MODEL. The acetylation sites identified in this study are indicated, colored in yellow to indicate an increased abundance in the stat phase, with gray indicating no significant change. (B) ClustalW2 alignment of *B. subtilis* MreB, MreBH, and Mbl with selected bacterial orthologs. The acetylated sites are indicated, color coded as described in panel A. (C and D) Distributional dot plots summarizing *B. subtilis* cell length (C) and width (D) measurements for wild-type (BD630), *mreBK240Q* (BD7587), *mreBK240R* (BD7619), and Δ*mreB* (BD3955) strains (*n* = 150 cells per strain) grown in the log or stat phase. The middle line (green, log; red, stat) in each plot represents the mean cell length or width. Statistically significant differences in length or width compared to the wild-type strain (Bonferroni-corrected *P* values) are marked with an asterisk.

10.1128/mSystems.00005-16.6 Figure S6  Proteins with discordantly regulated acetylated sites. Shown are the domain schematics of the remaining proteins with discordantly regulated acetylated sites (Fig. 5B). Acetylated residues are denoted by amino acid positions and color coded for downregulated (blue) or upregulated (yellow) relative change in the stat versus log phase. Protein domains were labeled with InterPro terms obtained from the UniProtKB. Download Figure S6, PDF file, 1 MB. Copyright © 2016 Carabetta et al. 2016 Carabetta et al. This content is distributed under the terms of the Creative Commons Attribution 4.0 International license.

We next examined the complete set of differentially acetylated proteins, grouping them by their known enzymatic functions and biological processes. Consistent with previous acetylome studies (19, 24–26, 28, 29, 32, 34–43, 56) and with our qualitative analyses above, differentially acetylated proteins were statistically enriched in metabolic, biosynthetic, and translation processes, largely represented by dehydrogenases, oxidoreductases, ligases, and ribosomal protein functions (Fig. 4D). Only 53% of the proteins were part of the metabolic network, and the remaining set of differentially acetylated proteins were associated with many other areas of bacterial physiology, including transport, DNA metabolic processes, and anatomical structure and morphogenesis (see Fig. S7 in the supplemental material). As these functions have not been linked to acetylation in bacteria, we further analyzed the relationships between the acetylated proteins in these processes. By generating functional and abundance-coded protein networks (Fig. 4E and F), we observed that the majority of the protein expression levels do not significantly change (see Table S2), the lone exception being the single-stranded binding protein SsbA, which increases expression in the stat phase (Fig. 4E). While we did not determine the absolute stoichiometry of acetylation, by directly measuring both relative protein abundance and relative acetylation abundance, we can make inferences about whether any changes in stoichiometry have occurred. Specifically, we determined that the protein abundances in these networks (Fig. 4E and F) were largely unchanged, while specific acetylations were differentially regulated. Therefore, we concluded that most of the acetylation abundance differences in these networks are bona fide changes in modification stoichiometry.

10.1128/mSystems.00005-16.7 Figure S7  GO biological process (BP) classification of differentially acetylated proteins. Differentially acetylated proteins (*n* = 273) were classified and clustered by their GO biological process ontologies (GO BP 03-10-2015) using the ClueGO cytoscape plugin (version 2.1.7). The node size reflects the relative number of genes assigned to each GO BP term. Download Figure S7, PDF file, 0.2 MB. Copyright © 2016 Carabetta et al. 2016 Carabetta et al. This content is distributed under the terms of the Creative Commons Attribution 4.0 International license.

To our knowledge, acetylation of a structural cytoskeletal protein has not been characterized for prokaryotes. Intriguingly, proteins involved in both the regulation of and the cytoskeleton itself (i.e., actin, and microtubules) are acetylated in eukaryotes (57). In addition, some of these acetylation modifications may have biological significance, such as in the stabilization of actin stress fibers (22) and promotion of the bundling of microtubules (58). MreB, an actin homolog, contains three acetylated residues, 240, 247, and 298; however, only K240 acetylation was significantly increased upon transition to the stat phase (Fig. 4F). In *B. subtilis*, MreB is an essential protein that is primarily responsible for regulating the diameter of the cell (59, 60) and for controlling peptidoglycan (PG) elongation (61–63). Since acetylation is likely to be a regulatory mechanism in controlling the eukaryotic cytoskeleton, we focused on the acetylation of the essential MreB in order to determine its potential regulatory role in cell shape determination in bacteria.

### MreB lysine 240 is involved in control of cell width and cell wall biosynthesis.

To gain insight into the possible role of MreB K240 acetylation, we first performed a structural analysis. No crystal structure for *B. subtilis* MreB has been determined, so we created a homology model using the *Caulobacter crescentus* structure (PDBe accession no. 4cze [59% sequence identity]). All three lysine residues are predicted to be surface exposed and are clustered to one face of the protein (Fig. 5A). Interestingly, K240 is well conserved in the Gram-positive organisms and is the most conserved of the three acetylation sites (Fig. 5B). The Gram-negative organisms and *Bacillus* paralog Mbl (MreB-like) contain an arginine residue at this position, implying that maintenance of a positive charge is important.

To assess the potential role of acetylation, we constructed glutamine (acetyl-mimic) and arginine (unmodified) single point mutations (64) at the *mreB* native locus. Depletion of MreB was achieved using a strain in which the native copy of *mreB* was deleted and the *mreBCD* genes were placed under inducible control*.* Since the main function of MreB is in the regulation of cell shape, we determined the length and width of 150 cells in both the log and stat phases. In the log phase, the wild-type cell length averaged 2.88 ± 0.55 µm (Fig. 5C). The K240R and *mreB-*depleted strain cell lengths were nearly identical at 2.75 ± 0.57 µm and 2.81 ± 0.64 µm, respectively. The only statistically significant difference was the length of the K240Q cells, in which the population mean was shorter, with an average of 2.55 ± 0.54 µm (*P* < 0.0001). As wild-type, K240R, and *mreB*-depleted cells enter the stationary phase, they get shorter (1.53 ± 0.28, 1.52 ± 0.29, and 1.52 ± 0.42 µm, respectively) on average (Fig. 5C). Again, the K240Q population was on average even shorter (1.41 ± 0.33 µm; *P* = 0.007). These data indicate that acetylation may have a minor impact on cell length, as the acetyl-mimic MreB showed a modest effect.

**FIG 6:**
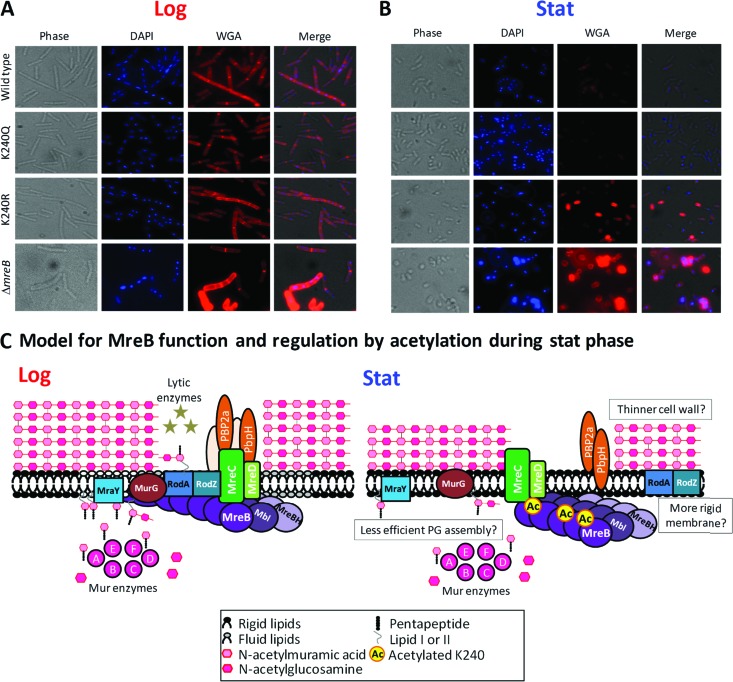
Acetylation mutants alter the peptidoglycan layer of the cell wall. (A and B) Phase-contrast and fluorescence microscopy of wild-type (BD630), *mreBK240Q* (BD7587), *mreBK240R* (BD7619), and *mreB*-depleted (BD3955) cells in the log (A) or stat (B) phase. Depicted are representative images from 2 independent experiments. Cell morphology was visualized by phase contrast, while cell surface saccharides were visualized by wheat germ agglutinin (WGA [red]). DNA was visualized by DAPI (blue). All exposure times were identical for WGA staining, except for the *mreB-*depleted log-phase images, where the exposure time was reduced 5-fold. (C) A hypothetical model for the role of MreB in coordination of cell wall assembly during log- and stat-phase growth. MreB is associated with its two paralogs (Mbl and MreBH), as well as the inner membrane complex of MreC, MreD, RodA, RodZ, MraY, MurG, and various penicillin-binding proteins (including PBP2a and PbpH, among others). In this model, MreB serves as an organizational scaffold to physically bring together cytoplasmic peptidoglycan (PG) precursor synthesis with extracellular PG assembly, leading to efficient cell wall growth. MreB may also associate with regions of increased membrane fluidity. During the stationary phase, acetylation of K240 increases significantly. As this residue does not lie in the regions required to make MreB filaments, we propose that it lies in an important interaction interface. Disruption of the interactions with any of MreB’s known interacting partners could lead to dissociation of the complex and a reduction in rate of PG elongation, formation of a thinner cell wall, and/or a change in membrane rigidity. These possibilities are not mutually exclusive and could lead to the shorter and thinner cells observed in the stationary phase.

We next assessed the impact of acetylation on cell widths (Fig. 5D). Interestingly, the K240Q cells were narrower (0.68 ± 0.09 µm; *P* < 0.0001) than the K240R or wild-type cells (0.75 ± 0.08 and 0.74 ± 0.10 µm, respectively) during the log phase, while the *mreB*-depleted strains were significantly wider (0.97 ± 0.13 µm; *P* < 0.0001), as expected (60, 65). These results suggest that MreB acetylation (K240Q) leads to cell width constriction, as the phenotype is evident during log phase when this residue is not normally acetylated. As wild-type cells enter into stat phase, they become narrower (0.59 ± 0.09 µm). Indeed, the acetylation mimic K240Q (0.56 ± 0.08 µm) was nearly identical to the wild type in cell width. In contrast, both K240R (0.68 ± 0.16 µm; *P* < 0.0001) and *mreB*-depleted (0.74 ± 0.17 µm; *P* < 0.0001) cells were significantly wider. During the stat phase, the observation that the acetyl-mimic mutation (K240Q) did not cause drastic cell width changes while the prevention of acetylation (K240R) led to cell widening is consistent with the fact that K240 was only acetylated during the stat phase. MreB has been implicated in the regulation of PG elongation, so we reasoned that acetylation of K240 may be an important regulatory event in modulating this function. To address this possibility, we stained the cell walls with wheat germ agglutinin (WGA), which binds the PG component *N*-acetylglucosamine. In the log phase, there was a slight reduction in staining in the K240Q mutant, while the K240R mutant appeared to be identical to the wild type (Fig. 6A). However, for the *mreB*-depleted cells, a large increase in WGA staining was evident (Fig. 6A). Upon entry into the stat phase, wild-type cells stain weakly with WGA, and the K240Q mutant stained even less intensely. The K240R cells showed a substantial increase in staining, but not as much as cells with *mreB* depletion (Fig. 6B). These data indicate that the inability to acetylate K240 leads to an alteration of the cell wall, likely affecting the PG. In addition to restricting cell width, the acetylation of lysine 240 may be important for altering cell wall structure as cells enter the stat phase.

## DISCUSSION

### Determinants of acetylation selectivity and the mechanism of acetylation.

We have determined that acetylation site selectivity is dependent upon factors beyond protein abundance (Fig. 2; see Fig. S2 in the supplemental material), most likely neighboring amino acids. Our analysis revealed a statistically significant enrichment for acidic residues in the neighborhood of acetylated lysines and a preference for tyrosine at the +1 position (Fig. 3B). Although it is possible that the identified sequence motifs only represent a subset of the total population of acetylation sites owing to differences in antibody specificity and affinity, our use of two antiacetyllysine antibodies probably served to decrease this potential bias (66). Our identified acetylation motif is in agreement with other bacterial data (24, 27–29, 32, 35, 36, 38, 40, 41, 43), while differing significantly from eukaryotic nuclear and cytoplasmic acetylation motifs. These tend to be enriched in aromatic and basic residues, while histone motifs are glycine rich (14, 21, 67–70).

In eukaryotic cells, the sequence motifs are thought to represent recognition signatures for the three families of identified KATs (71). In prokaryotes, the only known KATs belong to the GNAT family and specifically acetylate AMP-forming acyl-CoA synthetase enzymes at a conserved active site lysine (44, 72). This acetylation site, LP(R/K)TX(S/T)GKac(L/I)Q(K/R), does not resemble the motifs identified in this study, suggesting that AcuA in *Bacillus* is not responsible for global acetylation. Moreover, we identified a potential unique stat-phase-specific motif in which Asp and His were under- and overrepresented at position +1, respectively, compared to log-specific motifs (Fig. 3C to E). This motif suggests that there is specificity in the selection of acetylation targets, and it could potentially reflect the sequence specificity of unidentified enzymes that are active in the stat phase. Interestingly, a BLAST search of the *B. subtilis* genome revealed about 50 proteins with homology to the ubiquitous Gcn5 family of acetyltransferases (5, 6), suggesting that additional, uncharacterized protein acetylases may exist.

On the other hand, it is important to consider that many lysine acetylation events may be nonenzymatic. Emerging evidence has led to the proposal that in mitochondria (73, 74) and bacteria (34, 35, 43), a nonenzymatic mechanism contributes to global protein acetylation. In a basic environment, as in the mitochondrial matrix, lysine residues are relatively deprotonated, potentially serving as efficient nucleophiles for nonenzymatic acetylation. This is reminiscent of the reaction mechanism of the GNAT enzymes, which utilize a general base catalyst to deprotonate the target lysine residue, facilitating nucleophilic attack on the carbonyl carbon of the acetyl group in acetyl-CoA (6, 75). For example, the yeast KAT Gcn5p uses a conserved glutamate residue (E173) to deprotonate the target lysine residue (76). Additionally, a eukaryotic *N*^α^-acetyltransferase (Naa50p) depends on a tyrosine or histidine residue to act as a general base catalyst (77), which interestingly, were the other 2 amino acids significantly enriched at the +1 position in our identified motifs (Fig. 3). Unlike the mitochondrial lumen, *B. subtilis* maintains a cytoplasmic pH of ~7.4 (78), suggesting a less favorable environment for a nonenzymatic mechanism than in mitochondria. However, the local chemical environment of a target lysine residue would likely influence its nucleophilicity and may compensate for this difference. Thus, our identified motif may be an essential part of an autocatalytic mechanism for protein acetylation in *B. subtilis*, *Escherichia coli*, and human mitochondria.

For *E. coli* cells grown in minimal medium supplemented with glucose, global lysine acetylation was dramatically increased in the stat phase compared to the log phase, consistent with the increased levels of the metabolic intermediate acetyl-phosphate in the cell (34). Furthermore, a mutation in the enzyme that breaks down acetyl-phosphate (acetate kinase [encoded by *ackA*]) leads to an increase in the intracellular level of acetyl-phosphate and also increases global acetylation (34, 35, 43). From this work, Kuhn et al. proposed that global acetylation in *E. coli* occurs via a nonenzymatic mechanism, with acetyl-phosphate serving as the acetyl source rather than acetyl-CoA (35). However, this pattern is in stark contrast to our observations in *Bacillus*, which show a dramatic decrease in stat-phase protein acetylation (Fig. 1B and C). Although the reasons for the stat-phase decrease in *B. subtilis* are unknown, they may be due to differences in acetate metabolism and therefore levels of the acetylation donor, whether acetyl-CoA or acetyl-phosphate. We regard the likelihood of a nonenzymatic mechanism as an open question. If the acetylation occupancy were found to be very high at a given site, it would seem that the plausibility of an inherently slow nonenzymatic mechanism would be diminished.

### Roles of acetylation in protein regulation.

Although thousands of acetylation sites in several bacterial species have been catalogued, the functional relevance remains unknown for nearly all of these modification sites. Our analysis revealed proteins involved in important cellular pathways that were differentially acetylated in the two growth phases (Fig. 4D; see Fig. S7 in the supplemental material). Two interesting examples that had previously not been linked to regulation by acetylation are the DNA metabolism and the anatomical structure and morphogenesis clusters (Fig. 4E and F). In both cases, the acetylation patterns change, while the levels of the proteins themselves are relatively constant. It is possible that these acetylation events regulate protein function, as they often are located within or near known functional domains. For example, polynucleotide phosphorylase (Pnp) has two sites (K172 and K420) acetylated in the log phase that are predicted to be within exoribonuclease phosphorolytic domains 1 and 2, respectively. Another example is thioredoxin reductase (TrxB), which is acetylated during the log phase at two sites—one (K125) located 10 residues away from the active site and another (K195) within the NAD-binding domain.

An interesting subset of proteins had at least one site that was quantitatively discordant (Fig. 4B), going up or down in the stat phase while other acetylations in the same protein changed in the opposite direction. These discordant patterns hint at specificity in the rates of acetyllysine formation at different sites. If these rates are determined by nonenzymatic mechanisms, then they must be differentially affected by factors that change as cells move into the stat phase (e.g., protein folding or protein-protein interactions). Altogether, the diversity of functional networks that contain specific acetylation sites changing in abundance provides evidence for global regulatory mechanisms acting on different pathways.

### Does MreB acetylation regulate cell shape?

The phenotypes of the MreB-K240Q and -K240R substitution mutants are consistent with the idea that acetylation of this residue reduces the rate of PG synthesis. The K240Q cells are shorter and narrower in the log phase, show no phenotype in the stat phase, and stain more weakly with WGA, consistent with the exclusive detection of K240 acetylation in the stat phase (Fig. 4F, 5C and D, and 6A and B). Possibly, acetylation leads to a reduction in PG synthesis and a reduction in cell size. In the stat phase, the K240R cells are wider than normal, perhaps due to an inability to acetylate this residue (Fig. 5D). All of the identified MreB acetylation sites, including K240, lie in a surface-exposed region (Fig. 5A) that is not involved in the creation of an interface for interaction with other MreB molecules in the filament (79). Since MreB is believed to be a scaffold protein, this surface may be involved in interaction with other proteins or lipids. Indeed, MreB is known to interact with a number of proteins (Fig. 6C) that are essential for PG elongation, including both cytoplasmic and extracellular components of PG precursor synthesis and assembly (60, 62, 63, 65, 80–84). One proposed role for MreB is to coordinate PG elongation by guiding precursors from the cytoplasm to assembly sites on the surface of the cell (61, 62, 84). It was also recently discovered that MreB filaments are associated with lipid regions of increased fluidity, and MreB itself may be involved in the distribution of lipids and membrane proteins (63). In addition, the PG precursor lipid II has a strong preference for association with fluid membranes (85), which may represent a mechanism by which MreB organizes PG elongation. It is plausible that acetylation of K240 on a population of MreB molecules could disrupt or create new critical contacts made with any of these interacting partners that alter the rate of PG elongation, or some other property of the cell wall (thickness, composition, or membrane rigidity), in the stationary phase to restrict cell size. Taken together, the surface-exposed location of this residue and the observed phenotypes of K240 mutations suggest that this region of the protein may be involved in protein-protein or protein-lipid interactions and that acetylation may modulate these interactions. Although the importance of lysine acetylation is frequently studied using single point mutant substitutions of lysine to either glutamine, regarded as an acetyl-mimic, or arginine, which cannot be acetylated but retains a positive charge (64), caution is essential when interpreting the phenotypic consequences of these substitutions because the side chains of glutamine, acetyllysine, lysine, and arginine are chemically distinct. Nevertheless, this type of analysis has proven useful previously, as glutamine substitutions have been demonstrated to recapitulate the effects of acetylation *in vitro* (86, 87) and *in vivo* (87–90), although not for all proteins (91, 92).

In summary, our study demonstrates that acetylation is prevalent and regulated in *Bacillus subtilis* during the transition from the log phase to the stat phase. Acetylated proteins function in pathways that extend beyond the previously reported translation and central metabolism processes*.* Our finding that MreB is dynamically modified by acetylation during cell growth in a way correlating with cell size suggests a plausible role for regulation by acetylation.

## MATERIALS AND METHODS

### Bacterial strains, media, and growth conditions.

The *Bacillus subtilis* strains used in this study are listed in Table 1. *Bacillus* strains were constructed by transformation with selection for appropriate antibiotics (93). Minimal glucose medium was supplemented with 50 µg/ml histidine, leucine, and methionine. Antibiotics were added where appropriate, and the concentrations used were as follows: 5 µg/ml erythromycin (Ery), 100 µg/ml spectinomycin (Spc), and 5 µg/ml kanamycin (Kan). Bacteria were grown in Luria broth (LB) or minimal medium at 37°C with aeration, and growth was monitored in a Klett colorimeter. Stellar competent cells (Clontech) were used for cloning. Plasmids in *E. coli* were selected for and maintained in the presence of 100 µg/ml ampicillin (Amp).

**TABLE 1:** Strains used in this study

Strain	Relevant genotype	Source^a^
*B. subtilis*		
BD630	*his leu8 metB5*	Lab strain
BD3955	*his leu8 metB5 amyE*::*P_xyl_-mreBCD* Spc Δ*mreB*::Kan	This study
BD7587	*his leu8 metB5 mreB-K240Q*	This study
BD7619	*his leu8 metB5 mreB-K240R*	This study
*E. coli*		
ED1881	Stellar/pminimad2*-mreBCD* Amp	This study
ED1888	Stellar/pminimad2*-mreBCD-K240R* Amp	This study
ED1895	Stellar/pminimad2*-mreBCD-K240Q* Amp	This study

a The *P_xyl_*-*mreBCD* and Δ*mreB*::Kan constructs were kindly provided by R. Daniels and J. Errington.

### Plasmid and strain construction.

To construct the *mreB* point mutations at the native locus, we utilized the pminimad2 cloning strategy, as described previously (94). The *mreBCD* operon was amplified from BD630 genomic DNA using the primers 5pMM-mreB (5′-TGATTACGCCAAGCTTTGCTAGAGACCTTGGTATAGATCT [in which the underlined portion represents homology to the vector]) and 3pMM-mreD (5′-GTGAATTCGAGCTCGGTACCTCTTCACAATATTCACCTCAAC) and cloned into the HindIII and KpnI sites on plasmid pminimad2 using the In-Fusion HD cloning kit (Clontech), as per the manufacturer’s instructions. All oligonucleotides were provided by Eton Biosciences (Union, NJ). The plasmid pminimad2*-mreBCD* (pED1881) was used as a template to create the K240 variants. The plasmid was mutagenized using the Change-IT multiple mutation site-directed mutagenesis kit (Affymetrix), per the manufacturer’s instructions, using the primer 5mreBK240Q (5′-CTCACAGGTTTGCCGCAAACAATTGAAATTACA [with the mutagenized nucleotide underlined]) or 5mreBK240R (5′-CTCACAGGTTTGCCGAGAACAATTGAAATTACA). All plasmid constructs were confirmed by sequencing performed by Eton Biosciences. The resulting plasmids, pED1888 and pED1895, were used to transform BD630 to create the *mreB-K240R* (BD7587) and *mreB-K240Q* (BD7619) point mutations at the native locus, respectively. The resulting *Bacillus* strains were confirmed by sequencing.

### Western blotting.

A 50-ml culture of BD630 was grown in minimal glucose medium until the mid-log phase and stat phase, and 25 ml was pelleted at each time point. Extracts were prepared by resuspending the pellets in 1 ml STM (50 mM Tris-HCl, pH 8.5, 50 mM NaCl, 25% sucrose, 5 mM MgCl_2_), and the cells were lysed by sonication. Cellular debris was pelleted, and the protein concentration was determined from the resulting supernatants by Bradford assay (BioRad). Samples were mixed with cracking buffer (0.225 M Tris-HCl, pH 6.8, 50% glycerol, 5% sodium dodecyl sulfate [SDS], 0.05% bromophenol blue, 1% β-mercaptoethanol) and boiled for 10 min, and equal amounts of protein were loaded on a 12% Tris-Tricine gel. Proteins were then transferred to a Protran nitrocellulose membrane (Whatman), blocked in 5% bovine serum albumin (BSA), and probed using a mixture of a 1:1,000 dilution of 2 different anti-acetyllysine antibodies (PTM Biolabs and ImmuneChem) in 5% BSA, as per the manufacturer’s recommendation. A 1:5,000 dilution of purified goat anti-rabbit antibodies conjugated to peroxidase (Abcam) was used as a secondary antibody. Bands were visualized using the enhanced chemiluminescence ECL Prime kit (Amersham) and imaged using a MyECL imager (Thermo Scientific).

### Preparation of cells and cryogenic cell lysis.

BD630 was grown overnight at 30°C on multiple glucose minimal medium plates. Cells were scraped from plates, pooled, and used to inoculate 8 liters of liquid glucose minimal medium. Six liters of cells was grown at 37°C to mid-log phase in glucose minimal media or 2 liters was grown to the early stat phase. Cells were harvested, frozen as pellets in liquid nitrogen, and subjected to cryogenic cell lysis as described previously (95–97).

### Protein precipitation and trypsin digestion.

From the ground frozen cell powder, 0.1 g (previously estimated to be ~3 to 5 mg of protein) was resuspended in 2 ml of TEST buffer {100 mM Tris, pH 8.0, 2% SDS, 5 mM tris(2-carboxyethyl) phosphine [TCEP], 1 mM EDTA} that was previously heated to 95°C. Samples were sonicated and then centrifuged at 21,000 × *g* for 10 min at 20°C, and the resulting supernatants were heated to 70°C for 10 min for sample reduction. Proteins were alkylated by treatment with 10 mM chloroacetamide (final concentration) for 1 h at 37°C. The reaction was quenched by addition of 10 mM cysteine for 15 min at 37°C. After incubation, samples were cooled on ice, and 4 volumes of ice-cold acetone was slowly added, drop by drop. Tubes were incubated at −20°C for 1 h, and lysates were clarified by centrifugation at 3,000 × *g* for 10 min at 4°C. The resulting pellets were washed 3 times with 80% cold acetone and allowed to air dry. The dried pellets were resuspended in a 1-ml solution of 50 mM ammonium bicarbonate, 0.1% RapiGest surfactant (Waters), and 1 mM CaCl_2_. The protein concentration was determined using a Coomassie (Bradford) protein assay kit (Pierce), and input material was normalized. Precipitated proteins were digested with 50 µg of sequencing-grade modified trypsin (Promega) for 4 h at 37°C, and then a second 50 µg of trypsin was added, and the mixture was allowed to digest overnight. Trypsin was then inactivated by incubation for 5 min at 95°C.

### Enrichment of acetylated peptides.

The enrichment of acetylated peptides was carried out as described previously, with some modifications (98). The samples were acidified by addition of 1% (final concentration) trifluoroacetic acid (TFA) and incubated for 45 min at 37°C to cleave the RapiGest. The peptide solutions were centrifuged at 21,000 × *g* for 10 min at 20°C, and the pellets were discarded. The supernatants were split equally into 4 tubes, dried to completeness by vacuum centrifugation, and then each washed with 1 ml high-performance liquid chromatography (HPLC)-grade water and dried to completeness. This was repeated 3 times until no visible salt pellet remained. The lyophilized pellets were resuspended and combined in a total volume of 1.5 ml NETNA binding buffer (50 mM Tris-HCl, pH 8.0, 100 mM NaCl, 1 mM EDTA, 0.5% NP-40, 10% acetonitrile [ACN]). Centrifugation was performed at 21,000 × *g* for 10 min to remove any remaining insoluble material. From the supernatants, aliquots of ~50 µg of peptides were reserved for total cell protein analysis, and then a mixture of 50 µl of prepared pan acetyllysine antibody (PTM Biolabs) conjugated to protein A beads (Santa Cruz Biotechnology) and 50 µl of a commercial anti-acetyllysine antibody agarose (ImmuneChem) was added and allowed to incubate for 5 h at 4°C with rotary shaking. Conjugation of antiacetyllysine antibody to protein A agarose beads was carried out as described previously (98). The beads were collected by centrifugation at 1000 × *g* for 2 min and then washed 3 times with 1 ml of NETN buffer (50 mM Tris-HCl, pH 8.0, 100 mM NaCl, 1 mM EDTA, 0.5% NP-40), 3 times with ETN (50 mM Tris-HCl, pH 8.0, 100 mM NaCl, 1 mM EDTA), and finally 1 time with 1 ml HPLC water at 1,000 × *g* for 2 min at 4°C for each wash. Peptides were eluted from the beads with 150 µl of elution buffer (0.5% TFA, 5% ACN), repeated 3 times. Our enrichment efficiency was routinely between 25 and 40%. The pooled supernatants from acetyllysine enrichment were concentrated by vacuum centrifugation. Both acetyllysine supernatants and total cell digests were acidified to 1% TFA. SDB-RPS (Empore [3 M]) StageTips were used to desalt samples, and for total cell digests, peptides were separated by stepwise elution into three fractions, as previously described (99). The lysine enrichment and whole-cell digest analyses were repeated 3 independent times.

### Mass spectrometry analysis of acetyllysine enrichments and whole-cell digests.

Peptides (4 µl) from biological triplicates of acetyllysine peptide enrichments and corresponding whole-cell lysate digests from the log and stat phases were analyzed by nanoscale liquid chromatography (nLC) coupled online to either an LTQ Orbitrap Velos (acetyllysine) or XL (whole-cell) mass spectrometer (Thermo Fisher Scientific, Inc.). Peptides were loaded directly on column (PepMap C_18_ RSLC: 1.8 µm, 75 µm by 50 cm) and resolved by reverse-phase chromatography using a linear 3-h gradient of 4 to 40% buffer B (A, 0.1% formic acid in H_2_O; B, 97% ACN in 0.1% formic acid). Eluted peptides were analyzed by data-dependent acquisition (DDA) methods. Briefly, full MS spectra were acquired in the Orbitrap analyzer (resolution, 30,000; lock mass enabled for the Velos), from which precursors were selected for tandem MS (MS/MS) fragmentation. To increase the coverage and confidence of the identified acetylations, the complementary fragmentation techniques, collision-induced dissociation (CID [Top 15]) and higher-energy C-trap dissociation (HCD [Top 10]) were employed in two separate technical replicate DDA runs. HCD fragmentation provided signature immonium ion fragments diagnostic for acetyllysine. For whole-cell analysis, DDA CID (Top 10) fragmentation was employed. In all runs, the following instrument settings were used: Fourier transform MS (FTMS) and ion trap MS (ITMS) target values of 1E6 and 1E4 (Velos) or 5E3 (XL), respectively, and FTMS and ITMS maximum ion times of 500 and 100 ms, respectively. Dynamic exclusion was enabled with a repeat count of 1.

### Peptide identification and label-free protein quantification.

Thermo RAW instrument files for both acetyllysine enrichment and whole-cell data sets were processed together using MaxQuant (version 1.5.2.8) for peptide identification and label-free quantification. For the MaxQuant experimental design, the acetyllysine and whole-cell data sets were defined as separate parameter groups. MS/MS spectra were extracted using the default settings, and the database search was performed against the UniProt SwissProt/TrEMBL *Bacillus subtilis* (strain 168) sequence database (release 2015-02, 4,243 sequences), appended with common contaminants. The search parameters were configured identically for each parameter group, allowing for variable modification of oxidized methionine and protein N-terminal and lysine acetylation, as well as fixed modification of cysteine carbamidomethylation. Transferring of MS/MS sequencing events to nonsequenced features in other runs was enabled. However, to maintain the protein-level quantitative accuracy of the whole-cell data set, the label-free quantification (LFQ) algorithm (100), which employs mass recalibration and between-run peptide-level normalization, was performed separately within each parameter group. In addition, acetyllysine-containing peptides were excluded from protein quantification. To control false identification rates, PSM, protein, and site FDR were set at 1%, calculated based on matches to a reverse database. MaxQuant result files were imported into Perseus (version 1.5.1.6) for quantitative analysis, specifically acetyllysine peptides with their corresponding peptide intensity values, and protein groups with their corresponding LFQ abundance values (whole cell). Acetyllysine peptides were filtered to require (i) at least two valid intensity values in at least one group and (ii) a minimum PTM localization probability of 0.75. For whole-cell analyses, protein group assignments were filtered to require (i) at least two valid intensity values in at least one group and (ii) at least two sequenced razor + unique peptide identifications. After filtering, missing values were addressed by imputation based on a normal distribution using default settings. Differentially regulated acetyllysine sites and cellular proteins were determined using the Volcano plot function (*S*_0_ = 1; FDR, <0.05), computing FDR-corrected *P* values (*t* test) versus the magnitude of fold change (stat/log phase). Protein copy numbers were estimated based on 0.25 pg of protein per cell and using LFQ values normalized by the theoretical number of tryptic peptides (101). Processed and filtered data were exported to Microsoft Excel for further bioinformatics analysis.

### Bioinformatics and functional pathway analysis.

For GO analysis, primary accession numbers of proteins identified from whole-cell or acetyllysine peptide capture experiments were submitted to the Panther GO databases (www.pantherdb.org) or the ClueGO cytoscape plug-in (version 2.1.6) (102). The Panther GO analysis was used for statistical overrepresentation of individual biological processes, while ClueGO was used to generate biological process networks using ontology clustering. ClueGO was used to compare the biological processes shared between two lists of accession numbers and to generate GO biological process networks (both with grouping enabled, kappa score = 0.4). UniProt accession numbers were submitted to STRING (version 10) (103) to generate protein-protein functional networks. STRING networks were imported into cytoscape (version 3.2.1) (104) for overlay of quantitative data. All acetyllysine sites and neighboring amino acids were analyzed by the PhosphoSite motif and logo analysis tool (105). The *Caulobacter crescentus* (4cze.1.A, 59% identity) MreB structure was used as a template to create a homology model using SWISS-MODEL software (106). ClustalW2 (107) was used to generate the cladogram and sequence alignment.

### Statistical analysis.

Calculations of differences between actual versus expected lysine frequency were performed utilizing a comparison of proportions and applying a Bonferroni correction to *P* values for multiple comparisons (108) using Microsoft Excel 2010. The test of independence comparing log- and stat-phase acetyllysine distributions was performed similarly. All subsequent statistical analyses were performed using Stata/SE 13.1. While the utilization of Pearson’s correlation coefficient is more common when analyzing the correlation between variables, we wanted to look at a more general association between lysine number or protein abundance and number of acetylation sites. For this reason we used a Spearman rank correlation, since it is not limited to simple linear relationships and is also nonparametric (109). The distributional dot plots were constructed using Stata/SE 13.1. Differences between mean width and length were analyzed using a one-way analysis of variance (ANOVA) followed by *post hoc t* test. All *P* values displayed have been Bonferroni corrected to account for multiple comparisons.

### Microscopy.

*Bacillus* strains were grown in LB medium to the mid-log or stat phase. For cell length measurements, at each time point 500 µl of cells was harvested, and the cells were incubated for 5 min in 2 µg/ml propidium iodide (PI) on poly-l-lysine-coated slides, washed 10× with phosphate-buffered saline (PBS; 81 mM Na_2_HPO_4_ plus 24.6 mM NaH_2_PO_4_ plus 100 mM NaCl), and then mounted in Slowfade (Molecular Probes). For wheat germ agglutinin (WGA [Life Technologies]) staining, cells were attached to a poly-l-lysine-coated slide, incubated for 1 min in the presence of 2 mg/ml (final concentration) lysozyme, and then immediately washed three times in PBS. Cells were stained for 15 min with 2 µg/ml (final concentration) 4′,6-diamidino-2-phenylindole (DAPI) and 1 µg/ml WGA and then washed and mounted as described above. Microscopy was performed with an upright Nikon Eclipse 90i microscope outfitted with an Orca-ER digital camera (Hamamatsu) and a Nikon TIRF 1.45 NA Plan Neo-Fluor 100× oil immersion objective. The Volocity software package (version 6.3; PerkinElmer) was used for image acquisition and measurements. The exposure time for the WGA staining was 50 ms for every strain in both growth phases, with the exception of the *mreB* mutant in log phase, which was imaged for 10 ms. For individual cell measurements, the length and width of 150 cells were determined for each strain.
