# Supplementary material for: Temporal Regulation of the Bacillus subtilis Acetylome and Evidence for a Role of MreB Acetylation in Cell Wall Growth
Source: mSystems. 2016 May 31;1(3):e00005-16. doi: 10.1128/mSystems.00005-16 (PMC4927096; doi:10.1128/mSystems.00005-16)
Supplement: Figure S3 [file sys003162024sf3.pdf]

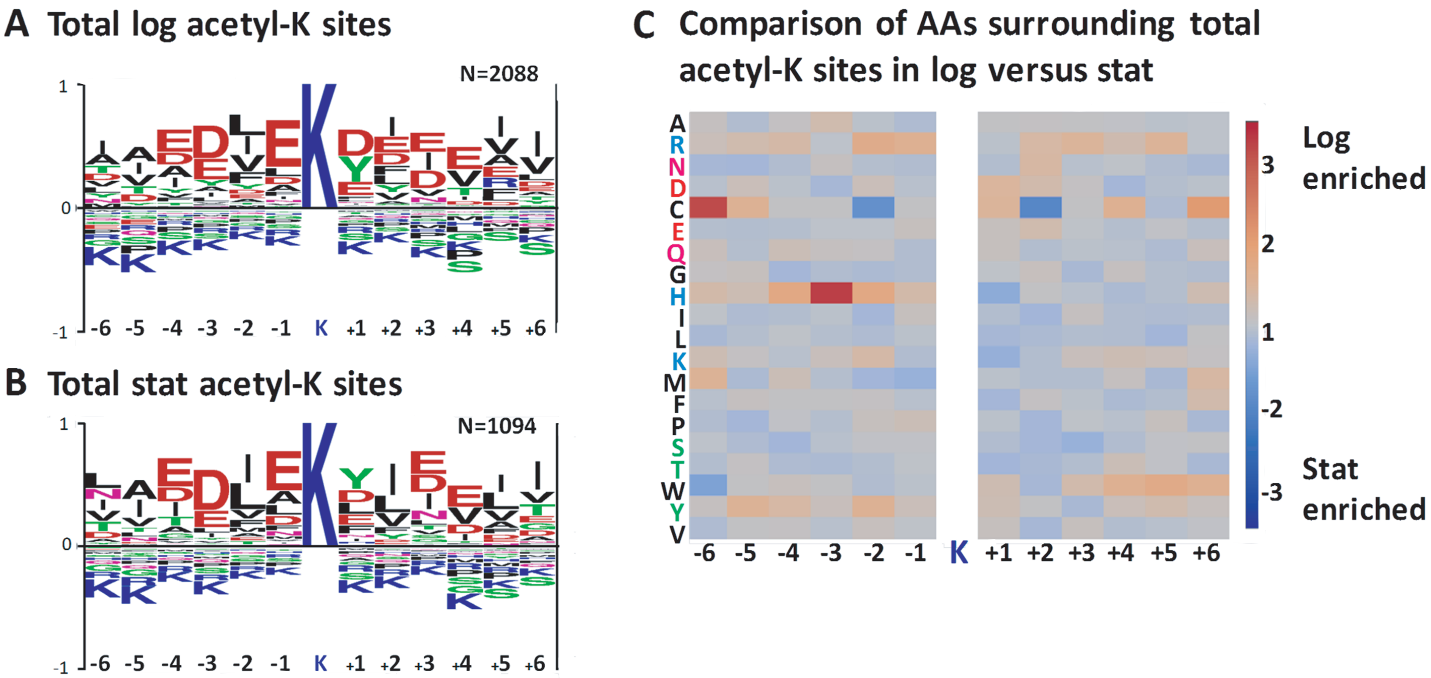
A Total log acetyl-K sites
N=2088
B Total stat acetyl-K sites
N=1094
-6 -5 -4 -3 -2 -1 K +1 +2 +3 +4 +5 +6
C Comparison of AAs surrounding total acetyl-K sites in log versus stat
A R N D C E Q G H I L K M F P S T W Y V
Log enriched
Stat enriched
3
2
1
-2
-3
