# Supplementary figures and images for: Temporal Regulation of the Bacillus subtilis Acetylome and Evidence for a Role of MreB Acetylation in Cell Wall Growth
Source: mSystems. 2016 May 31;1(3):e00005-16. doi: 10.1128/mSystems.00005-16 (PMC4927096; doi:10.1128/mSystems.00005-16)

## A Total protein abundance

(N = 1947)

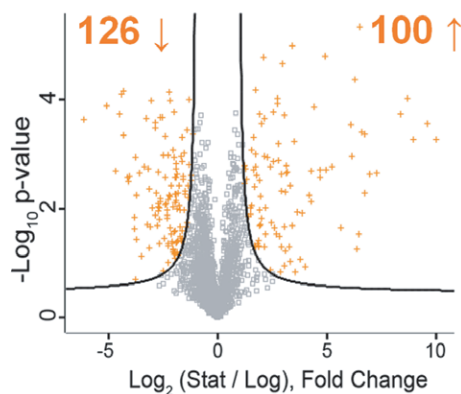

## B DNA mediated transformation network

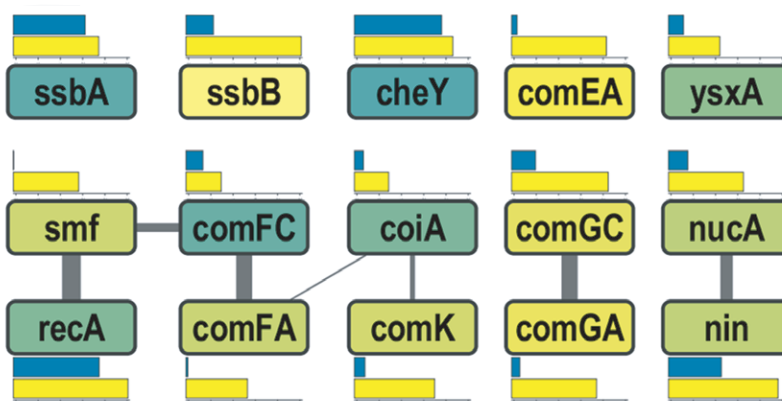

## C Translational network

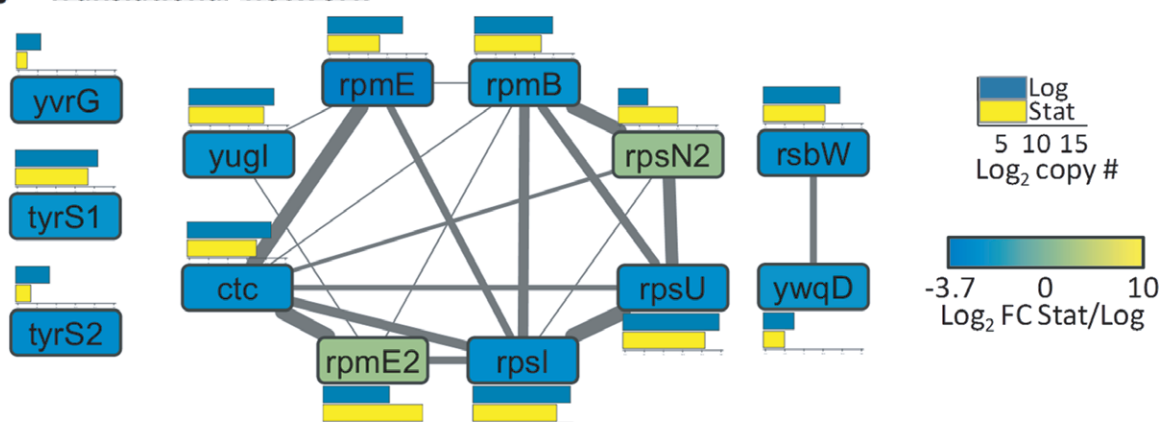

Supplement: Figure S4 [file sys003162024sf4.pdf]

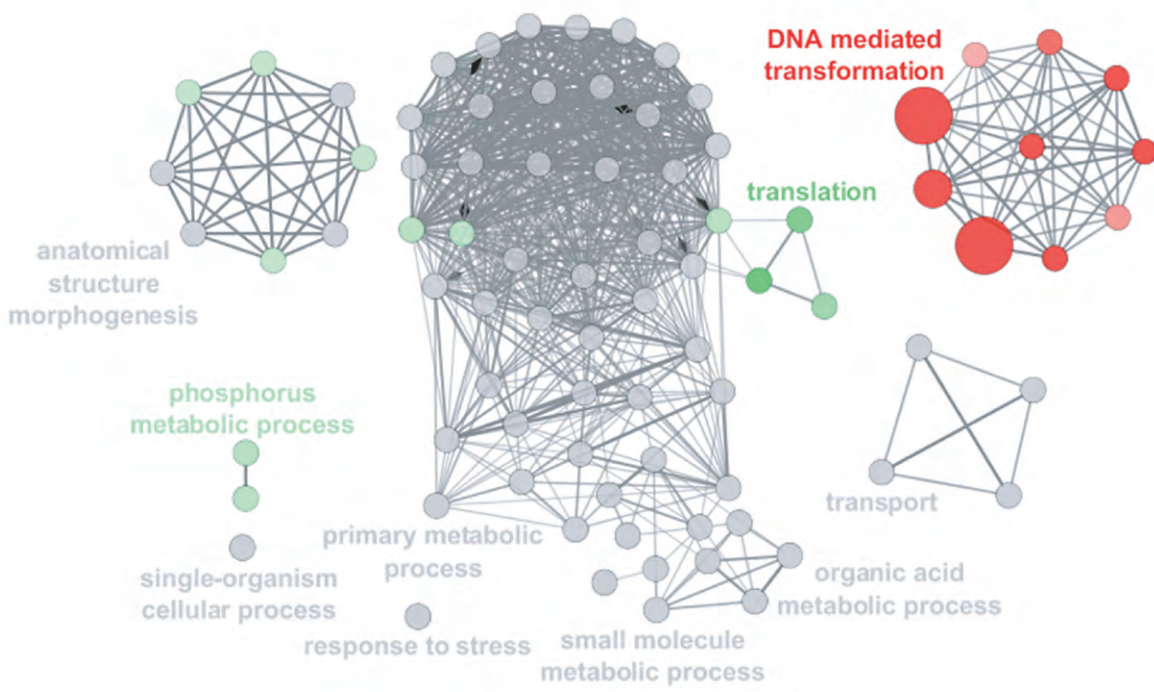

Supplement: Figure S5 [file sys003162024sf5.pdf]

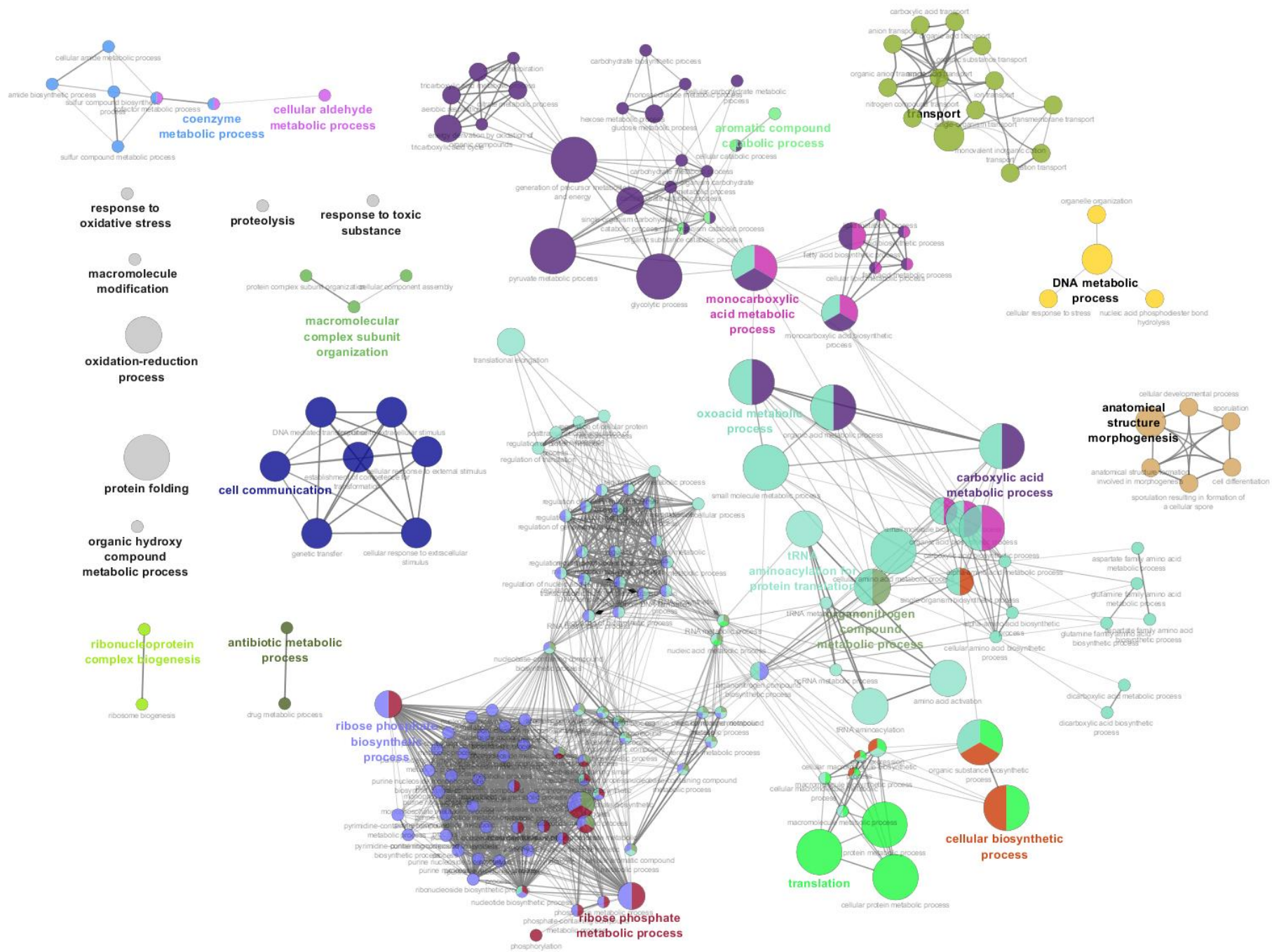

Supplement: Figure S7 [file sys003162024sf7.pdf]
