# Supplementary material for: Temporal Regulation of the Bacillus subtilis Acetylome and Evidence for a Role of MreB Acetylation in Cell Wall Growth
Source: mSystems. 2016 May 31;1(3):e00005-16. doi: 10.1128/mSystems.00005-16 (PMC4927096; doi:10.1128/mSystems.00005-16)
Supplement: Figure S6 [file sys003162024sf6.pdf]

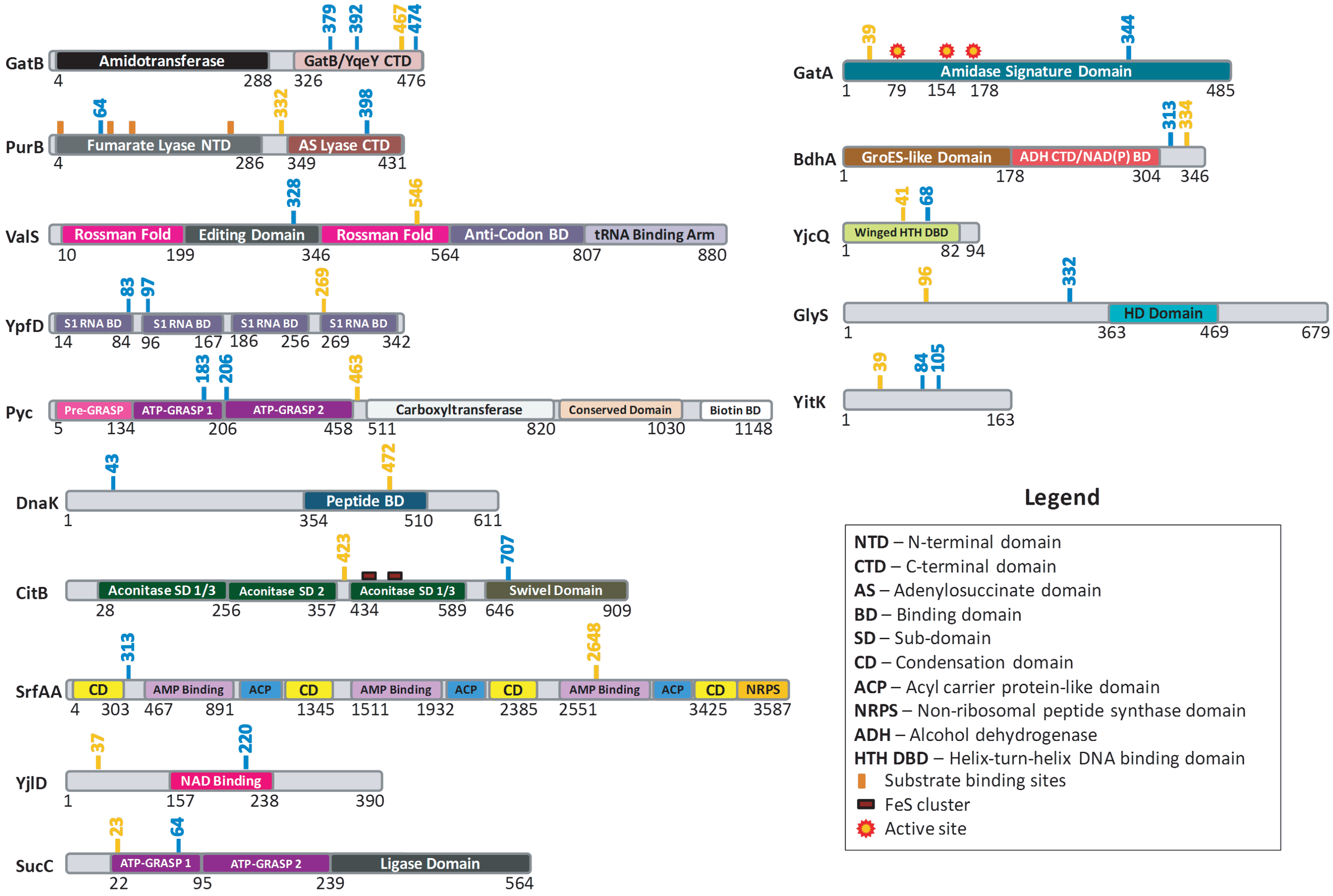
GatB
Amidotransferase
GatB/YqeY CTD
379 392 467 474
4 288 326 476
PurB
Fumarate Lyase NTD
AS Lyase CTD
64 332 398
4 286 349 431
ValS
Rossman Fold
Editing Domain
Rossman Fold
Anti-Codon BD
tRNA Binding Arm
328 546
10 199 346 564 807 880
YpfD
S1 RNA BD
S1 RNA BD
S1 RNA BD
S1 RNA BD
83 97 269
14 84 96 167 186 256 269 342
Pyc
Pre-GRASP
ATP-GRASP 1
ATP-GRASP 2
Carboxyltransferase
Conserved Domain
Biotin BD
183 206 463
5 134 206 458 511 820 1030 1148
DnaK
Peptide BD
43 472
1 354 510 611
CitB
Aconitase SD 1/3
Aconitase SD 2
Aconitase SD 1/3
Swivel Domain
423 707
28 256 357 434 589 646 909
SrfAA
CD
AMP Binding
ACP
CD
AMP Binding
ACP
CD
AMP Binding
ACP
CD
NRPS
313 2648
4 303 467 891 1345 1511 1932 2385 2551 3425 3587
YjlD
NAD Binding
37 220
1 157 238 390
SucC
ATP-GRASP 1
ATP-GRASP 2
Ligase Domain
23 64
22 95 239 564
GatA
Amidase Signature Domain
39 344
1 79 154 178 485
BdhA
GroES-like Domain
ADH CTD/NAD(P) BD
313 334
1 178 304 346
YjcQ
Winged HTH DBD
41 68
1 82 94
GlyS
HD Domain
96 332
1 363 469 679
YitK
39 84 105
1 163
Legend
NTD – N-terminal domain
CTD – C-terminal domain
AS – Adenylosuccinate domain
BD – Binding domain
SD – Sub-domain
CD – Condensation domain
ACP – Acyl carrier protein-like domain
NRPS – Non-ribosomal peptide synthase domain
ADH – Alcohol dehydrogenase
HTH DBD – Helix-turn-helix DNA binding domain
Substrate binding sites
FeS cluster
Active site
